# Injuries in Professional Roundnet (Spikeball) Athletes

**DOI:** 10.1055/a-2891-5869

**Published:** 2026-07-14

**Authors:** Ryan W. Paul, Ryan Park, Francis J. Sirch, Joelle Nyugen, Meghan E. Bishop, Kevin B. Freedman

**Affiliations:** 1 Orthopedic Surgery 24054 Monmouth Medical CenterLong BranchNew JerseyUnited States; 2 Neuroscience 3091 Lafayette CollegeEastonPennsylvaniaUnited States; 3 Department of Orthopedic surgery 24054 Monmouth Medical CenterLong BranchNew JerseyUnited States; 4 MarketingSpikeball Inc.ChicagoIllinoisUnited States; 5 Department of Orthopedic Surgery 387400 Rothman Orthopaedic InstitutePhiladelphiaPennsylvaniaUnited States

**Keywords:** injury, sports medicine, return to sport, return to play, shoulder, survey

## Abstract

This study aimed to evaluate training workload and injury history in specifically professional roundnet athletes. An anonymous survey regarding players’ injuries specific to roundnet was created with input from two premier roundnet athletes and two orthopedic surgeons. The 56 Spikeball Tour Series professional roundnet athletes from the 2023 season were contacted after the 2023 season to complete an anonymous injury history survey. Overall, 49 (88%) of the 56 professional roundnet athletes from the 2023 season were included in the study. Professional roundnet athletes had been competing in roundnet for a mean of 4 years and train 9.5 months per year. Players reported 82 roundnet injuries comprised of 43 (52%) upper extremity injuries, 24 (29%) lower extremity injuries, and 15 (18%) axial injuries. No reported injuries required surgical treatment. Upper extremity, lower extremity, and axial injuries had an average time to return to sport (RTS) of 1.8 months, 2.8 months, and 2.2 months missed, respectively. In conclusion, the upper extremity is the most commonly injured region in professional roundnet athletes, accounting for about half of roundnet injuries. Professional roundnet athletes most frequently experience significant acute injuries of the elbow and ankle, and significant chronic injuries of the shoulder and lower back.

## Introduction

**Fig. 1:**
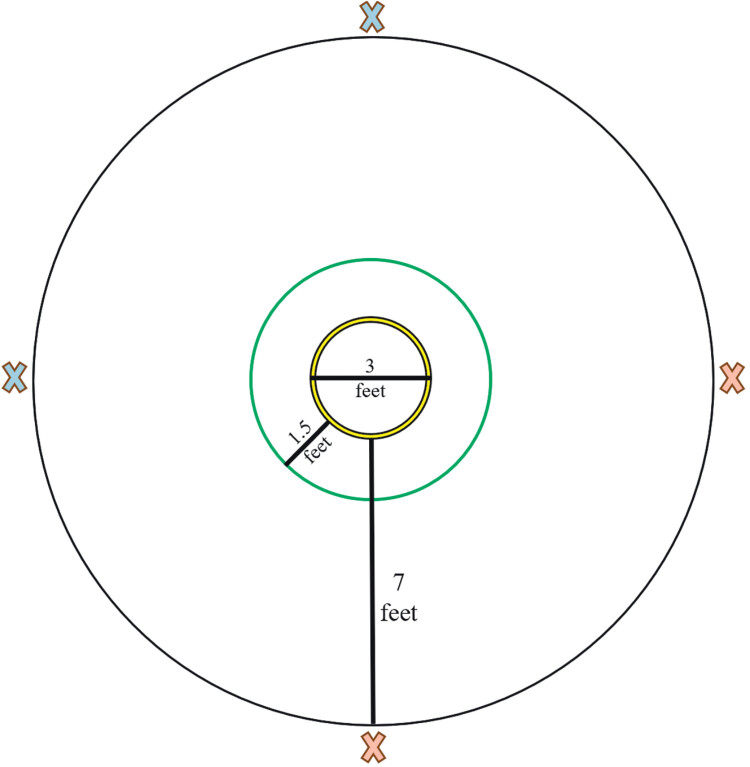
Layout of a competitive roundnet game as of 2023. Yellow circle is the 3-foot diameter roundnet set, green circle is the 4.5-foot diameter no-hit zone which the athlete cannot be standing inside when hitting the ball onto the net, and the black outer circle is the 8.5-foot diameter serving circle which the server and two other players adjacent to the server must be standing outside of during the serve. Blue and red X represent teams.

Roundnet (Spikeball) is a four-player ball sport where two teams are composed of two players each positioned around a circular net ( Fig. 1 ). Similar to volleyball, the ball is served to the opposing team and the opposing team may touch the ball up to three times before having to hit the ball onto the net, at which time it is conversely the other team’s turn to return the ball onto the net with three or less touches. The team that fails to return the ball onto the net within three touches loses the point.

People often play the sport casually; however, competitive tournaments have started in the past decade. The Spikeball Tour Series has become a leading tournament series in the sport, with 16 Open teams and 12 Women’s teams (each team has 2 players) earning the title “Pro” in the 2023 season. As the sport continues to gain popularity and grow in competitiveness, serving/hitting velocities, athletic requirements, and training volumes will likely increase, leading to an expected increase in chronic injuries. 1​ The sport also has several mechanisms predisposing athletes to acute injuries such as dives, falls, and collisions.

While current research on roundnet athletes is limited, a recent study by Paul et al. 1​ found that roundnet athletes experienced injuries most frequently involving the shoulder and ankle that often resulted in missed competition time. However, this study utilized online convenience sampling of players of all competitive levels, with only seven responses coming from the professional level where the imposed forces on the body and training workloads are likely the highest. Therefore, the purpose of this study was to evaluate training workload and injury history in specifically professional roundnet athletes.

## Materials & methods

### Participants

This cross-sectional study was exempt from Institutional Review Board (Thomas Jefferson University 22E.442) approval due to the minimal risk involved with anonymous survey completion and the anonymity of survey completion. This study was conducted with the support and help of Spikeball Inc. (Chicago, Illinois). All participants gave informed consent before taking part in this study. The 56 professional roundnet athletes from the 2023 Spikeball Tour Series season were eligible to participate in this study. Roundnet athletes earned the title of professional through a 6-month series of national roundnet tournaments, where the 16 male and 12 female teams with the most accumulated points earn professional status and exclusively compete in the national professional tournament. Athletes who were not professional roundnet athletes during the 2023 season, declined to participate, or did not complete the survey were excluded.

### Data collection

An anonymous RedCap survey (Vanderbilt University, Nashville, Tennessee) regarding players’ demographics, training workload, and self-reported prior injuries specific to roundnet was created with input from two premier roundnet athletes and two orthopedic surgeons. Professional roundnet athletes were contacted via email after the 2023 season by personnel within Spikeball to complete the anonymous injury survey.

The following demographic variables were collected: age, sex, height, weight, hand dominance, country, and roundnet training workload. Athletes then completed a self-reported roundnet-related injury history. Athletes were asked how many upper extremity, axial (head/neck/torso), and lower extremity injuries they have experienced from roundnet. They were then asked to describe each injury with specific prompts: a subjective description of the injury, the mechanism of injury, description of injury chronicity (acute: one specific moment of injury vs. chronic: no specific moment of injury but worsening with use), time until return to sport (RTS), whether operative treatment was required, and if so what the surgery was.

### Statistical analysis

Demographics, workload, and number of injuries were reported for the full cohort as well as isolated for male and female participants. Continuous variables were reported as mean±standard deviation, and categorical variables were reported as *n* (%). Rates of total injuries and injuries causing time away from sport were compared based on the joint injured. Rates of missing competition time and time until RTS was compared between upper extremity, axial, and lower extremity injuries. All statistical analyses were done using R Studio (Version 4.1.2, Vienna, Austria).

## Results

Overall, 49 (88%) of the 56 professional roundnet athletes from the 2023 season responded and were included in the study. Professional roundnet athletes had been competing in roundnet for a mean of 4 years, train 9.5 months per year, 3 days per week, and about 2 hours per training day ( Table 1 ). Players reported 82 roundnet injuries averaging 1.7 injuries per player. No reported injuries required surgical treatment. Details about each reported injury is available in **Supplementary Table A1** (available in the online version only).

**Table:** **Table 1** Demographics, workload, and number of injuries of 2023 roundnet professionals

Demographics			
Variable	Total (n=49)	Male (n=29)	Female (n=20)
Age (years)	24±2	24±2	24±3
Height (cm)	176±12	182±8	164±8
Weight (kg)	72±13	81±10	59±5
BMI	22±4	23±6	22±1
Right-Hand Dominant	46 (94%)	26 (90%)	20 (100%)
Country:			
USA	36 (73%)	22 (76%)	14 (70%)
Canada	5 (10%)	3 (10%)	2 (10%)
Austria	4 (8%)	2 (7%)	2 (10%)
Germany	2 (4%)	2 (7%)	0 (0%)
Switzerland	2 (4%)	0 (0%)	2 (10%)
Workload:			
Years of Competitive Roundnet	4.2±1.7 (1.5-9)	4.7±1.6 (3-9)	3.4±1.7 (1.5-
Months per Year Training	9.5±3.0 (2-12)	9.4±2.9 (2-12)	9.7±3.1 (2-
Days per Week Training	2.8±1.6 (1-6.5)	2.7±1.5 (1-5)	2.9±1.8 (1-
Hours per Day Training	2.3±1.0 (1-5.5)	2.2±1.2 (1-5)	2.3±0.8 (1-
Injuries			
**Variable**	**Total (n=49)**	**Male (n=29)**	**Female (n=20)**
Total Number of Injuries	1.7±1.5	1.8±1.6	1.5±1.2
Number of Upper Extremity Injuries	0.9±1.0	1.0±1.0	0.7±0.8
Number of Lower Extremity Injuries	0.5±0.6	0.4±0.6	0.6±0.6
Number of Axial Injuries	0.3±0.5	0.3±0.5	0.3±0.5

Workload is presented as mean±standard deviation (range).

**Fig. 2:**
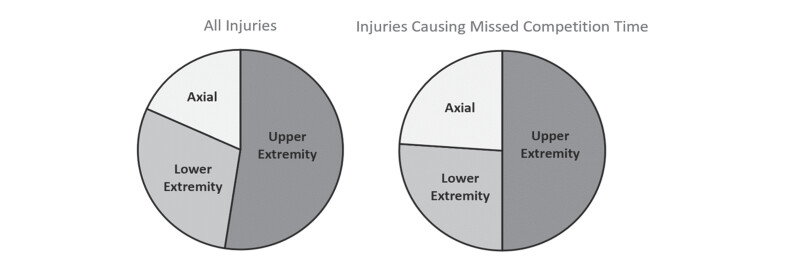
Comparison of region of injury for all injuries and specifically those that led to missed competition time.

The 82 total roundnet injuries were comprised of 43 (52%) upper extremity injuries, 24 (29%) lower extremity injuries, and 15 (18%) axial injuries ( Fig. 2 , Table 2 ). Most upper extremity injuries were of the shoulder (35%), wrist (26%), or elbow (23%), while most lower extremity injuries were of the ankle (54%) or knee (33%), and most axial injuries were of the lower back (67%). Mechanisms of injury to the upper and lower extremities were most often acute (52% and 67%, respectively) while injuries to the axial skeleton were most often chronic (74%). Regarding missed competition time, 53% of upper extremity, 50% of lower extremity, and 73% of axial injuries led to missed competition time. Upper extremity injuries had an average time until RTS of 1.8 months missed, while lower extremity injuries had an average 2.8 months missed, and axial injuries had an average 2.2 months until RTS.

**Table:** **Table 2** Injury location, mechanism, and outcome in 2023 roundnet professionals

Upper Extremity			
Variable	Total (n=43)	Male (n=30)	Female (n=13)
Location:			
Shoulder	15	9	6
Elbow	10	6	4
Wrist	11	10	1
Hand	7	5	2
Mechanism of Injury:			
Acute Dive	14	10	4
Acute Other	8	4	4
Chronic Serving	11	7	4
Chronic Other	10	9	1
Injury Caused Missed Competition Time	23/43 (53%)	16/30 (53%)	7/13 (54%)
Time Until Return to Sport (months)	1.8±1.5	2.0±1.7	1.4±0.6
Lower Extremity			
**Variable**	**Total (n=24)**	**Male (n=13)**	**Female (n=11)**
Location:			
Hip	2 (8%)	2 (15%)	0 (0%)
Knee	8 (33%)	4 (31%)	4 (36%)
Ankle	13 (54%)	6 (46%)	7 (64%)
Foot	1 (4%)	1 (8%)	0 (0%)
Mechanism of Injury:			
Acute Dive	5 (21%)	3 (23%)	2 (18%)
Acute Other	11 (46%)	5 (38%)	6 (55%)
Chronic Serving	4 (17%)	3 (23%)	1 (9%)
Chronic Other	4 (17%)	2 (15%)	2 (18%)
Injury Caused Missed Competition Time	12/24 (50%)	8/13 (62%)	4/11 (36%)
Time Until Return to Sport (months)	2.8±3.0	3.6±3.4	1.1±0.6
Axial			
**Variable**	**Total (n=15)**	**Male (n=9)**	**Female (n=6)**
Location:			
Neck	2 (13%)	0 (0%)	2 (33%)
Lower Back	10 (67%)	7 (78%)	3 (50%)
Ribs	2 (13%)	1 (11%)	1 (17%)
Head	1 (7%)	1 (11%)	0 (0%)
Mechanism of Injury:			
Acute Dive	1 (7%)	1 (11%)	0 (0%)
Acute Other	3 (20%)	2 (22%)	1 (17%)
Chronic Serving	1 (7%)	1 (11%)	0 (0%)
Chronic Other	10 (67%)	5 (56%)	5 (83%)
Injury Caused Missed Competition Time	11/15 (73%)	7/9 (78%)	4/6 (67%)
Time Until Return to Sport (months)	2.2±2.1	2.9±2.0	1.4±1.1

**Fig. 3:**
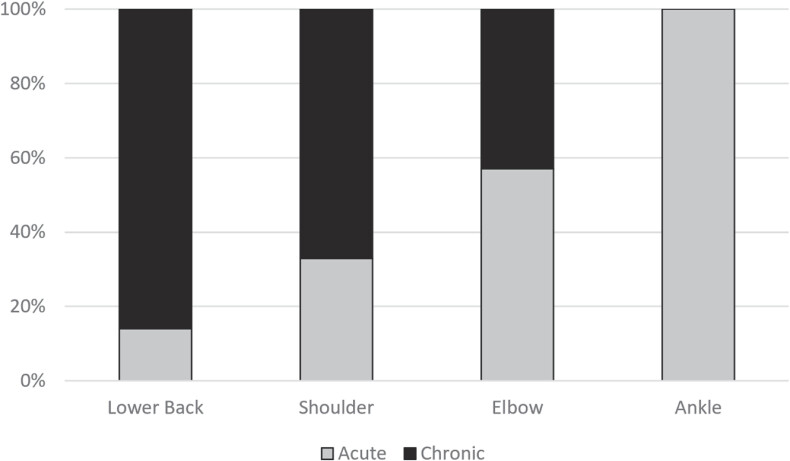
Mechanism of injury of the most common injury locations causing missed competition time. All joints with less than five injuries leading to missed competition time were excluded.

Injuries of the shoulder (22%) and ankle (16%) were the most common joints involved in all self-reported injuries, while the shoulder (20%), lower back (15%), and elbow (15%) were the most commonly injured joints that led to missed competition time ( Table 3 ). Chronic injuries that required missed competition time were more frequently of the lower back and shoulder while acute injuries that required missed competition time were more frequently of the elbow and ankle ( Fig. 3 ).

**Table:** **Table 3** Number of total injuries and injuries that caused missed competition time based on joint injured

Joint/Region	Number of total injuries ( *n* =82)	Number of injuries causing missed competition time ( *n* =46)
Shoulder	18 (22%)	9 (20%)
Ankle	13 (16%)	6 (13%)
Wrist	11 (13%)	4 (9%)
Lower back	10 (12%)	7 (15%)
Elbow	9 (11%)	7 (15%)
Knee	8 (10%)	3 (7%)
Hand	5 (6%)	3 (7%)
Neck	2 (2%)	1 (2%)
Ribs	2 (2%)	2 (4%)
Hip	2 (2%)	2 (4%)
Head	1 (1%)	1 (2%)
Foot	1 (1%)	1 (2%)

## Discussion

The purpose of this study was to evaluate training workload and injury history in specifically professional roundnet athletes. This study found that professional roundnet athletes had been competing in roundnet for a mean of 4 years and train about 6 hours per week for 9.5 months of the year. The 82 total roundnet injuries were comprised of 43 (52%) upper extremity injuries, 24 (29%) lower extremity injuries, and 15 (18%) axial injuries. Upper extremity, lower extremity, and axial injuries led to missed competition time 53%, 50%, and 73% of the time, respectively, and furthermore led to an average time until RTS of 1.8 months, 2.8 months, and 2.2 months, respectively. Injuries of the shoulder (20%), lower back (15%), and elbow (15%) were the most commonly injured joints that led to missed competition time.

A recent study by Paul et al. 1​ investigating injuries in roundnet athlete injuries across all competitive levels by distributing a REDCap survey through popular roundnet social media accounts. The survey assessed athlete demographics, training workload, and roundnet-related injuries. The authors found that out of 166 athletes, 86.1% ( *n* =143) of athletes reported at least one injury during their playing career. 1​ There were 279 total injuries reported across the 166 athletes, with 67% of injuries causing missed competition time. 1​ The average time until RTS was 2 months. 1​ Additionally, 10 (3.6%) injuries required surgery which caused athletes to miss an average of 7.2±2.7 months of play. 1​ The injuries that required surgery include two anterior shoulder dislocations, posterior shoulder instability, unspecified shoulder labral tear, two anterior cruciate ligament tears, Achilles tendon tear, meniscal tear, herniated intervertebral disc at L4-L5, and an unspecified lower vertebral disc rupture. 1​

When comparing the findings by Paul et al. 1​ regarding the non-elite athletes to the findings on professional roundnet athletes in the current study, both non-elite and professional roundnet athletes experienced a similar rate of injury (1.6 injuries per non-elite athlete vs. 1.7 injuries per professional athlete) despite noticeable differences in the years of roundnet experience (1.9 years non-elite vs. 4.2 years professional). Both non-elite and professional athletes experienced injuries most frequently of the upper extremity (professional: 52% vs. non-elite: 48%) and also injured the shoulder and ankle joints most frequently. Unlike the non-elite athletes who underwent six surgeries for roundnet injuries (4.6% of total non-elite injuries), the professional athletes evaluated in the current study had no surgeries to report. This finding may be because athletes who experience injuries that require surgery are less likely to successfully rehab and return to their same or better level of sport, thus limiting them from earning the professional title, while other athletes who were previously professional may actively be rehabilitating from a surgery during the 2023 season.

As the complexity of the sport of roundnet increase, the authors involved in roundnet have seen new peak levels of serving, hitting, and defense, increasing clinicians’ necessity to understand the strains that occur on the upper and lower extremities increases. For professional roundnet athletes, serving has evolved where hitting harder serves with both hands has found the top professional finding success. Because of this, increase strain on the upper extremity potential has led to these athletes experiencing overuse injuries. This can be seen in chronic overhead workload contributes to risk of injury by baseball and volleyball athletes 3​ 4​ ​5​ .

The authors have also seen diving in the sport of roundnet become increasingly important for professional roundnet athletes, heavily impacted by the introduction of the no-hit zone in 2021. The no-hit zone is a circular area 1.5 feet (0.46 meters) around the net where the player is not allowed to stand inside of when hitting the ball onto the net. Because of this, players often dive over the no-hit zone while hitting to optimize the angles for hitting available to them, with every dive coming with a risk of acute injury when landing. Another factor that may increase injury rates in professional roundnet athletes is the duration of tournaments, with tournaments often starting around 9:00 AM and athletes competing until they are eliminated from contention 5–10 hours later, depending on their success throughout the playoff bracket. Such prolonged competition times increase the risk of both acute and chronic injury, with fatigue drastically increasing injury risk in overhead athletes 6​ . Future studies evaluating fatigue in roundnet athletes can help clarify whether the effects of fatigue on injury risk can be generalized to the roundnet population.

In a study of 660 elite handball players, it was found that use of shoulder injury prevention programs reduces the prevalence of shoulder problems in elite handball athletes by 28% 7​ . Similarly, a randomized controlled trial on youth baseball players found that injury prevention programs decrease shoulder and elbow injuries from 3.1 injuries per 1000 athlete-exposures to 1.7 injuries per 1000 athlete-exposures 8​ . These studies highlight the importance of shoulder strengthening to help limit injuries, which may be generalized to the serving aspect of roundnet as well. A randomized controlled trial evaluating the effects of a bodyweight neuromuscular warm-up on lower limb injuries in elite female basketball players found that bodyweight neuromuscular warm-up was effective in reducing risk of lower limb injury in female players 9​ . While future research regarding injury prevention programs will be helpful to optimize injury prevention in roundnet, roundnet athletes would benefit from injury prevention recommendations from other sports in the meantime.

Overall, clinicians should be aware that almost half of reported injuries in professional roundnet athletes occur in the upper extremity, with chronic injuries of the lower back also common. Until further research regarding injury prevention protocols in roundnet athletes is completed, roundnet athletes and clinicians can extrapolate injury prevention recommendations from other sports and injury prevention programs. For roundnet athletes or clinicians preparing an injury prevention program, the authors recommend several aspects be incorporated in any and all programs. Due to the frequency of chronic shoulder injury, athletes should perform resistance training of the rotator cuff 2–3 days per week, as well as both general stretching of the shoulder and targeted internal rotation stretching such as the sleeper stretch and cross body adduction stretching. Roundnet athletes would also benefit from ankle stability training, with movements such as single-leg balancing, calf raises, and resisted ankle inversion/eversion with resistance bands being feasible exercises to help decrease the risk of ankle sprain. Finally, due to the frequency of lower back pain, roundnet athletes would benefit from both neutral spine core exercises (forward and side planks, glute bridges) and resisted rotational movements (med ball tosses, half-kneeling cable rotations).

While this study helps provide initial information regarding the injuries in roundnet, there is much potential for future research in this sport. First of all, future prospective studies should be designed to more accurately track injuries in roundnet athletes, with formal injury diagnosis from a sports medicine clinician further improving these future studies and potential takeaways. This prospective study design will also allow researchers to report exposure-based injury rates, which will more accurately report the true risk of injury. Also, future studies evaluating roundnet athletes before vs. after a tournament can help identify the patterns of fatigue in these athletes, which can help improve injury prevention strategies. Lastly, future trials that randomize roundnet athletes to varying injury prevention programs can help clinicians understand which injury prevention programs are best for this specific athletic population.

### Limitations

This study has several limitations. First, all injuries and workloads were self-reported from the roundnet athletes, which increases recall bias and limits diagnostic precision, as shown by some injuries described vaguely such as “shoulder pain” and “wrist injury”. Future studies that assess workload and injury prospectively would benefit from incorporating internal and external load measures to better assess workload and identify injury risk factors. Also, the injuries were reported as absolute counts per athlete instead of normalized to sport exposure, limiting the assessment of injury risk. Future studies would benefit from tracking training workload closely and reporting injuries via exposure-based metrics (i.e., injuries per 1,000 training hours). Plus, functional outcomes after injury were not assessed in this study. It is possible that these injuries had effects to athlete activities of daily living and roundnet performance that were not captured in the current study. Additionally, due to the self-reported and retrospective nature of the current study design, this study was not able to evaluate associations between potential risk factors (player demographics, workload, clinical measures, etc.) and injury.

## Conclusion

The upper extremity is the most commonly injured region in professional roundnet athletes, accounting for about half of roundnet injuries. Professional roundnet athletes most frequently experience significant acute injuries of the elbow and ankle, and significant chronic injuries of the shoulder and lower back. Roundnet athletes who are training often and competing for professional status may benefit from stretching and strengthening of the shoulder, lower back, and core in an effort to decrease their risk of common roundnet injuries.

## Ethical approval information, institution(s) and number(s)

Thomas Jefferson University #22E.442

## Supplementary Material

Supplementary Material Zusatzmaterial
