# Supplementary material for: Injuries in Professional Roundnet (Spikeball) Athletes
Source: Sports Med Int Open. 2026 Jul 14;10:a28915869. doi: 10.1055/a-2891-5869 (PMC13460297; doi:10.1055/a-2891-5869)
Supplement: Supplementary file 1 [file 10-1055-a-2891-5869_28971867.pdf]

Supplemental material: Injuries in Professional Roundnet (Spikeball) Athletes

Authors: Ryan W. Paul MD MBA, Ryan Park BS, Francis J. Sirch MD, Joelle Nguyen BA, Meghan E. Bishop MD, Kevin B. Freedman MD MSCE

Appendix Table A1. Description of all injuries and missed competition time.

Injury Description	Missed Competition Time
**Upper Extremity**	
Anterior shoulder pain	No
Constant shoulder pain	Yes - 2 weeks
Posterior shoulder pain	Yes - 1 month
Shoulder bursitis	No
Shoulder impingement	Yes - 2 months
Shoulder impingement	No
Shoulder impingement	Yes - 1 month
Shoulder injury	No
Shoulder overuse injury	Yes - 1 month
Shoulder overuse injury	Yes - 1 month
Shoulder pain	No
Shoulder pain	No
Shoulder subluxation	Yes - 2 months
Shoulder subluxation	Yes - 1 month
Torn labrum	Yes - 2 months
Constant elbow pain	Yes - 2 months
Constant elbow pain	No
Elbow injury	Yes - 6 months
Elbow injury	Yes - 1.5 months
Elbow injury	No
Elbow hyperextension injury	Yes - 1 week
Elbow hyperextension injury	Yes - 2 months
Elbow hyperextension injury	Yes - 2 months
Elbow hyperextension injury	Yes - 2 months
Lateral epicondylitis	No
Lateral epicondylitis	No
Medial epicondylitis	Yes - 6 months
Chronic wrist pain	No
Chronic wrist pain	No
Jammed wrist	No
Wrist injury	No
Wrist injury	Yes - 3 months
Wrist injury	Yes - 2 weeks
Wrist injury	Yes - 1 month
Wrist pain	No
Wrist pain	No
Fractured phalanx	No
Fractured phalanx	Yes - 1.5 months
Fractured phalanx	Yes - 1 month
Fractured phalanx	Yes - 1 month

Fractured phalanx	No
Fractured thumb	No
Jammed finger	No
**Lower Extremity**	
Hip pain	Yes - 2 weeks
Torn hip adductor muscle	Yes - 3 months
Constant knee pain	No
Constant knee pain	No
Knee	No
Knee hyperextension injury	Yes - 2 months
Knee injury	Yes - 9 months
Knee hyperextension injury	Yes - 1 month
Knee injury	Yes - 9 months
Knee injury	No
Meniscal tear	No
Patellar instability	Yes - 2 weeks
Torn patellar cartilage	No
Ankle fracture	Yes - 2.5 months
Ankle sprain	Yes - 2 months
Ankle sprain	Yes - 2 months
Ankle sprain	Yes - 1 month
Ankle sprain	Yes - 1 month
Ankle sprain	No
Ankle sprain	No
Ankle sprain	No
Ankle sprain	No
Ankle sprain	No
Shin splints	No
**Axial**	
L4/L5 herniated disc	Yes - 6 months
Overuse lower back injury	Yes - 5 months
Overuse lower back injury	Yes - 3 months
Overuse lower back injury	Yes - 1 month
Overuse lower back injury	No
Overuse lower back injury	Yes - 3 months
Overuse lower back injury	No
Overuse lower back injury	Yes - 1 month
Overuse lower back injury	No
Overuse lower back injury	Yes - 3 months
Cervical radiculopathy	Yes - 1 month
Cervical radiculopathy	No
Rib contusion	Yes - 2 weeks
Rib impinging on nerve	Yes - 3 weeks
Concussion	Yes - 1.5 months
